# Improving the safety and tolerability of local anaesthetic outpatient transperineal prostate biopsies: A pilot study of the CAMbridge PROstate Biopsy (CAMPROBE) method

**DOI:** 10.1177/2051415818762683

**Published:** 2018-03-05

**Authors:** D Thurtle, L Starling, K Leonard, T Stone, VJ Gnanapragasam

**Affiliations:** 1 Academic Urology Group, University of Cambridge, Cambridge, UK; 2 Department of Urology, Addenbrooke’s Hospital, Cambridge, UK; 3 Cambridge Urology Translational Research and Clinical Trials, University of Cambridge, Cambridge, UK; 4 Medical Physics and Clinical Engineering, Addenbrooke’s Hospital, Cambridge, UK

**Keywords:** Prostate cancer, transperineal biopsy, diagnostic intervention, patient safety, service delivery

## Abstract

**Objectives:**

The aim of this study was to pilot the use of a bespoke device (CAMPROBE, the CAMbridge PROstate Biopsy) to enable routine outpatient free-hand local anaesthetic (LA) transperineal prostate biopsies.

**Materials and methods:**

The CAMPROBE prototype was designed and built in our institution. Men on active surveillance due prostate resampling were invited to have a CAMPROBE biopsy as an alternative to repeat transrectal ultrasound-guided prostate biopsies (TRUSBx) as part of an approved trial (NCT02375035). Biopsies were performed using LA infiltration only, without sedation or additional analgesia. Patient-reported outcomes were recorded at day 0 and 7 using validated questionnaires and visual analogue scales (VAS). Complications were recorded prospectively.

**Results:**

Thirty men underwent biopsies with a median of 11 cores taken per procedure (interquartile range 10–12). There were no infections, sepsis or retention episodes. Haematuria and haematospermia occurred in 67% and 62% of patients, which are similar to rates reported for TRUSBx. Mean VAS for pain (0–10 scale) was less than 3 for every part of the procedure. All 30 men described the procedure as tolerable under LA. In total, 26/30 (86.7%) men expressed a preference for a CAMPROBE procedure over TRUSBx and a further 3 (10.0%) would have either.

**Conclusions:**

In this small pilot study, the CAMPROBE device and method appears to be a safe, simple and well-tolerated out-patient transperineal replacement for TRUSBx. A major new National Institute for Health Research grant will allow its further development from a prototype to a single use, low-cost disposable device ready for multi-centre testing.

**Level of evidence:**

1b: individual cohort study.

## Introduction

Prostate cancer (PCa) is the commonest male cancer, representing 25% of all male neoplasms in the UK.^1^ The mainstay of histological diagnosis remains a transrectal ultrasound-guided prostate biopsies (TRUSBx). Over 1 million TRUSBx are performed annually in the US and Europe.^2^ However, these are associated with unacceptably high risks of infection, with fevers or chills reported in over 10% and severe sepsis in 1–2%.^3,4^ Costs associated with UK hospitalisation episodes for biopsy-related sepsis alone are estimated to be £7–11 million annually.^5^ These costs and risks will be amplified by increasing referral trends, projected to rise by two-thirds by 2030, and by worrying rises in antibiotic resistance of up to fourfold.^6,7^ Additionally, the transrectal approach has recognised drawbacks in undersampling the anterior and apical aspects of the gland.^8^ It is clear that the TRUSbx approach is untenable as a long-term option as a diagnostic procedure.

Transperineal (TP) biopsy (TPBx) has significantly lower infective risks as it traverses the sterilised perineum and in fact predates TRUSBx.^9,10^ Techniques using a perineal brachytherapy template grid under general anaesthesia (GA) or a free-hand ‘fan technique’ under local anaesthesia (LA) are both well established, although significant regional variations in practice exist.^11^ The use of GA and specialised equipment for grid-based TPBx limit its utility as a routine replacement for TRUSBx. Attempts at doing grid-based biopsies without GA have reported use of 50–60 ml of LA, additional analgesia and/or significant patient pre-preparation, which is not compatible with routine out-patient or office-based practice.^12,13^

The free-hand LA TPBx technique is appealing as it requires only two perineal punctures and no specialist equipment. However, the procedure is not standardised nor commonly performed in UK centres. We sought a way to enable wider uptake of LA TPBx as a viable cost-effective and routine replacement for TRUSBx by developing the CAMbridge PROstate Biopsy (CAMPROBE). The CAMPROBE (patent pending) is a cannulated TP access system based on the co-axial concept but bespoke to the context of prostate biopsies.^14^ The integrated device allows for synchronous device insertion and LA infiltration under ultrasound guidance, negating the need for separate punctures, nerve blocks or sedation. Once in position, standard 18G core-needle biopsies can be taken through the retained cannula. No specialised equipment except a couch suitable for lithotomy and a linear array ultrasound probe are required. Here, we report clinical and patient-reported outcomes from our pilot trial of the prototype device.

## Materials and methods

### CAMPROBE design and manufacture

**Figure 1.**
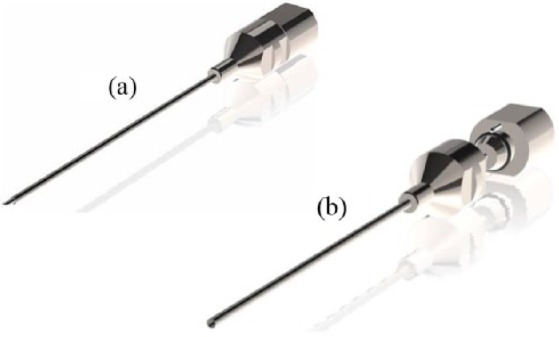
Diagrams demonstrating the prototype CAMPROBE (CAMbridge PROstate Biopsy) device used within this pilot study (patent pending). The integrated needle allows synchronous local anaesthetic infiltration during insertion (a). The central needle disarticulates from the surrounding cannula (b) to allow 18G prostate biopsies.

The CAMPROBE was designed towards the aim of producing a simple device to facilitate TP biopsies under LA, with procedures performed by a single operator with 1 assistant. The device (Figure 1) features an integrated needle to which a standard syringe can be attached for LA delivery. This is sheathed within a co-axial blunt-ended cannula long enough to penetrate from perineal skin to prostate capsule. The proximal end of the sheath was designed as a taper to permit easy introduction of a standard biopsy needle. The device was designed and manufactured in collaboration with the Clinical Engineering Innovation team (Cambridge University Hospitals National Health Service Foundation Trust), the design and manufacture met the requirements of the medical devices directive for in-house manufactured devices, and this was assured within the departmental International Organization for Standardization 13485-certified design processes. The design process followed an iterative healthcare design process developed by the Engineering Design Centre at Cambridge University (https://www-edc.eng.cam.ac.uk/research/healthcaredesign/).

### Clinical cohort

Following the innovation phase, we commenced the prospective development phase (Idea, Development, Exploration, Assessment, Long-term follow up (IDEAL) stage 2a) by piloting the prototype CAMPROBE device in an initial group of patients.^15^ Following trial approval and registration (NCT02375035) and ethical approval (Research Ethics Committee (REC) reference: 14/EE/1172), men aged 18–85, who were already awaiting a prostate biopsy and had undergone previous transrectal biopsies, were recruited from a prostate cancer monitoring (active surveillance) programme. As this pilot trial was designed to primarily test safety and tolerability, we excluded men with suspicious multi-parametric magnetic resonance imaging (mpMRI) lesions who needed image-guided biopsies.

### Procedure

**Figure 2.**
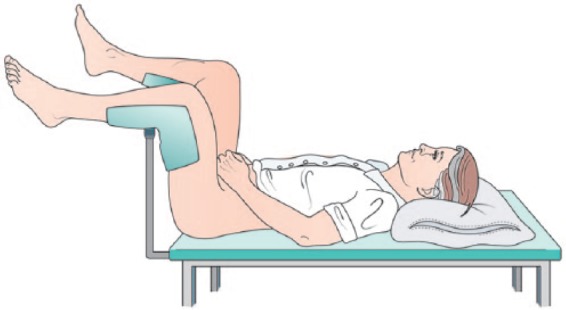
The patient is placed in a modified lithotomy position, with men supporting their own genitalia away from the operative field.

**Figure 3.**
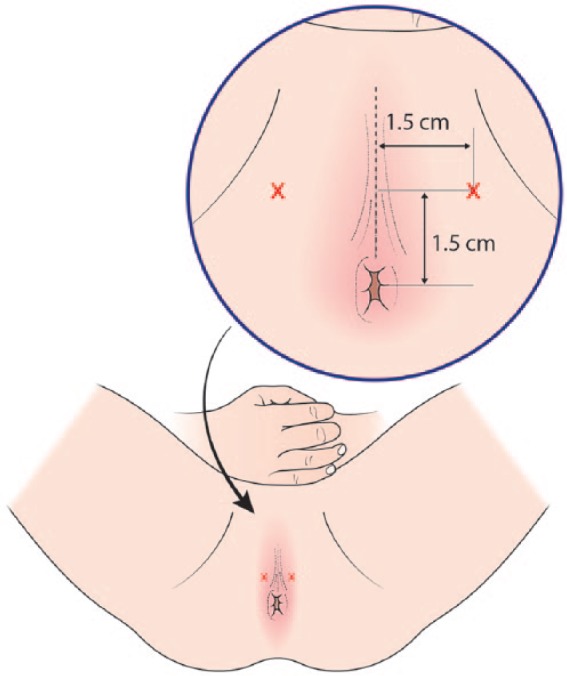
After skin preparation and draping, two points are marked 1.5 cm anterior and lateral to the anal verge and perineal midline.

**Figure 4.**
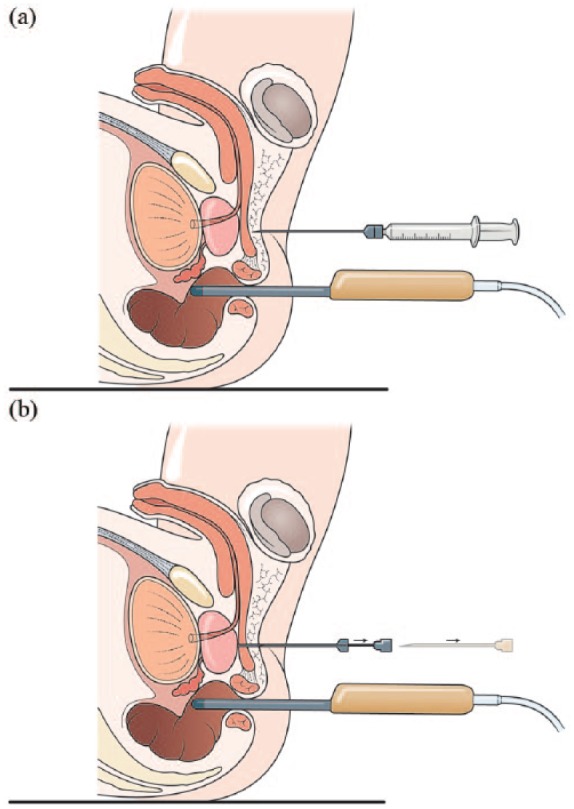
Sagittal diagrams demonstrating (a) CAMPROBE (CAMbridge PROstate Biopsy) insertion under ultrasonic guidance with synchronous local anaesthetic infiltration and (b) disarticulation of the integrated needle leaving the co-axial access sheath adjacent to the prostate.

**Figure 5.**
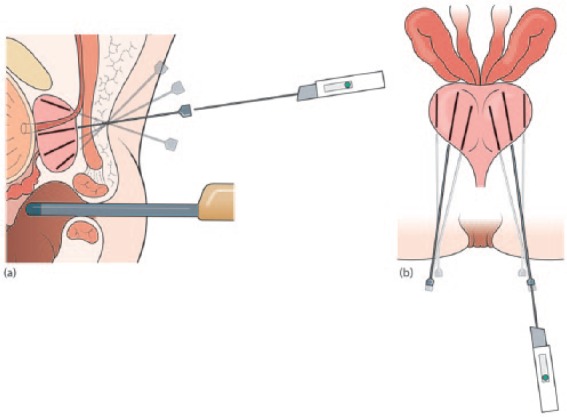
Sagittal (a) and axial (b) diagrams showing 18G biopsies being taken (using a BARD® biopsy device with 22 cm needle) through the retained CAMPROBE (CAMbridge PROstate Biopsy) cannula using a fan technique.

All procedures were undertaken in a clinic outpatient room. A single surgeon (VJG) performed the biopsies with the assistance of one nurse or health care assistant. All men received a pre-biopsy dose of ciprofloxacin 500 mg and had self-administered a glycerol suppository a few hours before the procedure. The patient was positioned in a modified lithotomy position on a perineal suturing couch with legs on holders (Figure 2). The perineal area was prepared with betadine or chlorhexidine wash. Two points were marked 1.5 cm above the anal verge and 1.5 cm on either side of the midline (Figure 3). At each marked site, 0.5 ml of 1% lignocaine was injected to numb the skin. The CAMPROBE was assembled and attached to a 5 or 10 ml syringe filled with 1% lignocaine (Figure 4(a)). A linear array ultrasonic transducer was placed in the rectum and the prostate and perineal tissue visualised. Starting on the right, the CAMPROBE was inserted under vision with LA delivered to sub-cutaneous tissue and deep perineal muscle synchronously as the device was advanced. As the device approached the prostate capsule, about 2–3 ml of LA was delivered to the peri-prostatic space. Once in position, the syringe and integrated needle was removed leaving the co-axial access sheath in place (Figure 4(b)). Through this sheath, standard 18G biopsies were taken (using a BARD® biopsy device with a 22 cm needle) from the posterior and anterior prostate, and mid and peripheral zones (a maximum of six on each side) using a fan approach (Figures 5(a) and 5(b)). If there was any further discomfort, the integrated needle was replaced and further LA injected without the need for a further skin puncture. In total, between 5–10 ml of lignocaine/side was required depending on the patient’s tolerability. The ultrasonic probe was rotated and angled to visualise the needle direction and biopsy site for each acquisition. In this respect, the thicker CAMPROBE cannula permitted improved visualisation of the biopsy needle direction. This was repeated on the contralateral side with a total procedure time of between 15–30 min. Once the CAMPROBE is removed, 2–3 min of perineal pressure is applied followed by light dressings to each puncture site. Post-biopsy, patients were managed in the standard fashion after a TRUSBx and given three days of post-biopsy oral antibiotics.

### Outcomes analysis

The primary objective was to assess patient-reported outcomes and tolerability of the procedure. Secondarily, we sought to assess the safety of the procedure by assessing incidence of infections or biopsy-related healthcare encounters. Evaluation was achieved using questionnaires adapted from the Prostate Biopsy Effects (ProBE) study, embedded within the ProtecT trial.^3^ All questionnaires underwent review and were approved by the assigned REC. Men self-reported pain, comfort, embarrassment and dignity on a four-point Likert-type scale immediately after and 7 days following CAMPROBE biopsy. Discomfort from each stage of the procedure and attitudes towards rebiopsy were assessed using a 0–10 visual analogue scale (VAS). The complete questionnaires are available in the online supplementary files. Complications or adverse events were self-reported at day 7, with the severity of complications assessed on a four-point scale from ‘none’ to ‘major’. For analysis, adverse events were classified according to the National Cancer Institute’s toxicity criteria, which ranges from 0 (where no adverse event is reported) to grade 4 (death as a result of the biopsy).^16^ Data were collated and analysed anonymously using Stata 14 (Texas, USA). There was no requirement to impute missing data.

## Results

### Study cohort

In total, 34 men were recruited to the study. Of these, 30 underwent the CAMPROBE procedure. Three men were unable to adopt the lithotomy position due to hip abduction problems and one man was unwell with an arrhythmia prior to biopsy. Median age and PSA was 71.5 years and 5.3 ng/ml respectively. There were no device failures or adverse events during the procedure. All men had undergone at least one previous TRUSBx (Table 1). All procedures were completed within 30 minutes. In total, 334 biopsy cores were taken (median 11, interquartile range 10–12). Pathological results were as expected in this low-risk cohort, with 13 (43.3%) men found to have cancer in one or more biopsy cores and four men (13.3%) pathologically upgraded as a result of the biopsy.

**Table 1.** Baseline patient characteristics of men undergoing CAMPROBE (CAMbridge PROstate Biopsy) (*n* = 30).

	Median	Range	Unit
Age	71.5	49–77	years
Prostate-specific antigen	5.3	0.72–36.9	ng/ml
	*n*	%	
Gleason grade:			
6	27	90.0	
7	3	10.0	
Reason for biopsy:			
Active Surveillance	30	100.0	
Number of previous biopsies:			
1	10	33.3	
2	15	50.0	
> 3	5	16.7	

### Patient-reported outcomes

Across the cohort, mean VAS scores for discomfort, on a 0–10 scale, for the initial digital rectal examination, scanner probe insertion, LA injection and biopsy-taking were 2.04 (SD 1.9), 2.97 (SD 1.9), 2.67 (SD 2.2) and 1.83 (SD 1.2) respectively (Table 2). No one reported the biopsies to be the most painful part of the procedure. No procedures were abandoned due to pain. In total, 29/30 men (96.7%) scored the procedure overall to be 4/10 or less for pain, with all 30 men describing the procedure as tolerable under LA. In addition, 27/30 men (90.0%) rated the CAMPROBE biopsy superior or equivalent to a transrectal biopsy. Overall, 20 and 9 men reported they would have ‘no problem’ and only a ‘minor problem’ with undergoing a repeated CAMPROBE biopsy, respectively (Table 3), and 26/30 (86.7%) men would prefer a CAMPROBE procedure over a transrectal biopsy for their next biopsy and a further 3 (10.0%) would have either.

**Table 2.** Summary of visual analogue scores for questions relating to the CAMPROBE (CAMbridge PROstate Biopsy) procedure, asked immediately after the procedure (*n* = 30).

Question	Score range	Mean (SD)	Median (interquartile range)
How much discomfort did the initial prostate examination (finger in the back passage) cause you?	0–9	2.04 (1.87)	2 (1–3)
How much discomfort did the insertion of the scanner probe cause you?	0–9	2.97 (1.96)	3 (2–4)
How uncomfortable was the presence of the probe in your back passage?	0–9	2.93 (1.96)	3 (2–4)
How much discomfort did the injection of local anaesthetic cause you?	0–9	2.67 (2.20)	2 (1–3)
How much discomfort did the actual taking of the biopsies with the needle cause you?	0–9	1.83 (1.18)	2 (1–3)

**Table 3.** Patient responses to questions on how painful or embarrassing they found the procedure, and how they would feel about another CAMPROBE (CAMbridge PROstate Biopsy) procedure if it was necessary (*n* = 30).

	Early (day of biopsy)				Late (7 days after biopsy)			
	Not at all	A little	Somewhat	A lot	Not at all	A little	Somewhat	A lot
Overall, how painful did you find the whole procedure?	4	19	6	1	2	22	5	1
Overall, how embarrassing did you find the whole procedure?	22	8	0	0	20	9	1	0
	Not a problem	Minor problem	Moderate problem	Major problem	Not a problem	Minor problem	Moderate problem	Major problem
How much of a problem would you have about having another CAMPROBE procedure?	23	5	2	0	20	9	1	0

### Clinical complications

There were no peri-procedural complications with all men discharged after the procedure. No men required hospital readmission in the week following their biopsy, including zero episodes of urinary tract infection or systemic sepsis. One man (3.3%) reported seeing his general practitioner as a result of pain from the biopsy and was prescribed painkillers. Twenty men (66.7%) reported visible haematuria by day 7 and 12 men reported haematospermia amongst a cohort of 19 who reported an ejaculation in the week following biopsy (63.2%). Two men (6.7%) reported blood in their stools that persisted for 4 and 5 days respectively. Median duration of haematuria, when present, was three days, no one required medical attention for bleeding and no one reported a bleeding complication as more than ‘a minor problem’. Two men (33.3%) reported alterations in urinary function in the week following the biopsy, with increased frequency and increased nocturia both reported; however, no one reported this to be more than ‘a moderate problem’ and no one sought medical attention. There were no episodes of urinary retention. Rates of fever, shivering or infection, haematochezia and biopsy-related health encounters were all much lower in our cohort compared to the ProBE study (Table 4).^3^ Haematuria and haematospermia rates were very similar.

**Table 4.** Patient-reported outcome measures at seven days post-procedure: comparing the CAMPROBE (CAMbridge PROstate Biopsy) procedure to reported outcomes following transrectal ultrasound-guided prostate biopsies (TRUSBx) in the ProBE study (*n* = 30).^3^

	Reported within CAMPROBE	%	For which you sought medical attention (%)		Reported within ProBE	%
*Since the procedure have you had*:						
Pain at the area of the biopsy?	12/30	40.0	1/30	3.3	425/1089	39.0
Fever?	0/30	0.0	0/30	0.0	128/1090^a^	11.7
Shivers?	1/30	3.3	0/30	0.0	135/1089	12.4
Retention of urine?	0/30	0.0	0/30	0.0	Not reported	
Visible haematuria?	20/30	66.7	0/30	0.0	693/1085	63.9
Blood from the back passage?	2/30	6.7	0/30	0.0	354/1076	32.9
Blood in the semen?	12/19	63.2	0/19	0.0	645/747	86.3
Biopsy-related health encounter?	1/30	3.33	-	-	134/1147	11.7

## Discussion

These early promising results from this pilot study suggest proof-of-principle that the CAMPROBE device and method for LA free-hand TP-biopsy approach is feasible, well tolerated by patients and preferable to transrectal biopsy for the vast majority of men. Amongst the ProtecT study TRUSBx ProBE cohort, 213/1085 (19.6%) reported that having a repeat transrectal biopsy would represent a moderate/major problem when asked at seven days.^3^ This compares to only 1/30 (3.3%) reporting a moderate problem with having another CAMPROBE procedure. We report a very low complication risk, importantly with zero episodes of infection or sepsis, and biopsy-related health encounters of any type (3.3%) were also considerably lower than the 11.7% reported following TRUSBx within the ProBE study.^3^ Both of which, if confirmed in larger trials, would have major patient safety and cost-reduction implications.

Because the CAMPROBE is an integrated device, LA is delivered under vision, which allows precise deployment of LA and hence reduced dosages. In addition, the free-hand approach makes the probe position much more comfortable for men, negating the need for anal sphincter relaxants or rectal LA gel. These facts make the CAMPROBE method much safer as an outpatient technique with minimal staffing (akin to the standard TRUSBx). The method exploits already existing equipment and consumables in any urology unit and will thus be easy to adopt. Future device costs are also projected to be low and on a par with current costs for disposable needle guides used for TRUSBx.

In 2015, a meta-analysis reported no difference in cancer detection between free-hand TP biopsy and transrectal biopsy.^17^ However, to our knowledge, this technique has not been widely adopted in the UK or elsewhere.^18^ Most studies have performed free-hand biopsies by simply inserting the biopsy needle into the perineal skin directly. In our view, this can be technically challenging due to the flexibility of the unsupported biopsy needle and the need for repeated skin passes. Certainly, the co-axial approach has been shown to significantly reduce patient discomfort from free-hand TP biopsies.^14^ Our method also goes further in reducing the need for separate punctures to deliver LA by integrating the LA delivery needle. The device also simplifies the procedure for the operating surgeon, with the device able to reach the prostate directly and an ergonomic design with a wide proximal funnel for simple single-handed biopsy-taking.

Limitations to this pilot study exist. As with any questionnaire-based study, limitations exist with regards to recall bias, particularly when comparisons are made to previous transrectal biopsy, which may have occurred many months earlier. However, the questionnaire response rate of 100% is a particular strength of the study. Completed VAS scores from 30 men is significantly higher than comparable publications drawing similar conclusions.^12^ Secondly, in this study we have focussed on men without targetable lesions on magnetic resonance imaging (MRI) as we did not want to potentially compromise histological detection. However, the technology could already be used for cognitive image-guided biopsies and no difference in clinically significant cancer detection has yet been demonstrated between cognitive and MRI-USS fusion techniques in either a comparative trial or systematic review of the relevant literature.^19,20^ Indeed, in a recent paper, Yaxley *et al.* reported no difference in either overall or clinically significant cancer detection rates in men having either image-guided in-bore MRI biopsies, TP grid-based biopsies or cognitively targeted TRUSBx.^21^ In this context, the CAMPROBE technique would also be preferable to transrectal biopsy due to the ability to access and sample the whole prostate gland. Image-guided techniques for use with free-hand TP biopsy are also likely to be clinically available in the very near future, with the technology rapidly developing, and two recent small studies demonstrating feasibility alongside the free-hand technique and suggesting superiority to systematic biopsy alone in detecting significant cancer.^22,23^ Further to this, we found that after device insertion there was no association with increasing numbers of biopsies taken and patient discomfort. Thus, we foresee no issues with obtaining both targeted biopsies and systematic sectoral biopsies using our method. In contrast, recent reports of grid-based TP approaches under LA for cognitive image-guided biopsies have only performed targeted biopsies, presumably to try and limit patient discomfort.^12^

The focus of this pilot study was to assess safety and tolerability rather than tumour detection. However, comparisons with recently published cohorts performing rebiopsy in a similar context provide reassurance that our method does not compromise tumour detection. For example, repeat 12-core sectoral systematic biopsy detected PCa in 56% of men on active surveillance (AS) with a negative mpMRI in a recent study from Johns Hopkins, Baltimore.^24^ In terms of detecting pathological tumour upgrade, 13.3% of men were upgraded on CAMPROBE systematic biopsy. This is very similar to the 11% reported amongst those with no MRI lesion in a similar study from Memorial Sloan Kettering, New York.^25^ Tumour detection is clearly an important outcome, and will be thoroughly assessed in future clinical testing.

As this was a pilot feasibility study, we do interpret our results with caution as we have small numbers, a lack of randomisation and no direct comparison to other biopsy types, including with respect to cancer detection. However, these will be addressed in a future multi-centre, large-volume (IDEAL stage 3) study. To facilitate this, we have recently secured a major ‘Invention for Innovation’ Product Development Award from the National Institute for Health Research (NIHR), which recognises the potential beneficial impact of this technique as a replacement for TRUSBx. Prospective NIHR funding over the next few years will enable the commercialisation of the CAMPROBE and transition from our surgical steel prototype to a disposable, CE-marked, mass produced single-use device. This will further reduce costs and increase accessibility to the device and technique, potentially accelerating its introduction into widespread clinical practice. The future scope for benefit is therefore potentially enormous and this innovation could have a major and critical impact for patients worldwide. Undoubtedly, there is a learning curve with the free-hand LA TP method as it involves dual hand co-ordination, which is different from the discipline of TRUSBx. However, free-hand TP biopsy is a well-known technique, with a number of publications suggesting it is a skill that can be acquired relatively quickly by a competent clinician.^22,26^ Of note, inability to tolerate the lithotomy position may be an issue in a small proportion of men, with 3/34 (8.8%) in our cohort unable to tolerate the position, predominantly due to hip discomfort.

In summary, TRUSBx for suspected PCa exposes men to excessive risk of infections and sepsis and contributes to an increasing crisis in antibiotic resistance. The CAMPROBE is a simple, cheap but disruptive technology that appears to be a good outpatient LA alternative, using the safer TP route. The simplicity and standardisation of the procedure should allow widespread uptake, with significant safety and cost benefits for health service delivery. Our current NIHR-funded work to develop a cheap, disposable version for the device will accelerate the journey to clinical practice.

## Supplemental Material

URO762683_questionnaire_1 – Supplemental material for Improving the safety and tolerability of local anaesthetic outpatient transperineal prostate biopsies: A pilot study of the CAMbridge PROstate Biopsy (CAMPROBE) method Click here for additional data file. Supplemental material, URO762683_questionnaire_1 for Improving the safety and tolerability of local anaesthetic outpatient transperineal prostate biopsies: A pilot study of the CAMbridge PROstate Biopsy (CAMPROBE) method by D Thurtle, L Starling, K Leonard, T Stone and VJ Gnanapragasam in Journal of Clinical Urology

## Supplemental Material

URO762683_questionnaire_2 – Supplemental material for Improving the safety and tolerability of local anaesthetic outpatient transperineal prostate biopsies: A pilot study of the CAMbridge PROstate Biopsy (CAMPROBE) method Click here for additional data file. Supplemental material, URO762683_questionnaire_2 for Improving the safety and tolerability of local anaesthetic outpatient transperineal prostate biopsies: A pilot study of the CAMbridge PROstate Biopsy (CAMPROBE) method by D Thurtle, L Starling, K Leonard, T Stone and VJ Gnanapragasam in Journal of Clinical Urology

## Supplemental Material

URO762683_questionnaire_3 – Supplemental material for Improving the safety and tolerability of local anaesthetic outpatient transperineal prostate biopsies: A pilot study of the CAMbridge PROstate Biopsy (CAMPROBE) method Click here for additional data file. Supplemental material, URO762683_questionnaire_3 for Improving the safety and tolerability of local anaesthetic outpatient transperineal prostate biopsies: A pilot study of the CAMbridge PROstate Biopsy (CAMPROBE) method by D Thurtle, L Starling, K Leonard, T Stone and VJ Gnanapragasam in Journal of Clinical Urology

## Notes

This video shows how the CAMPROBE procedure is undertaken step by step as well as the necessary equipment and preparation needed. For any queries please email the team and contact details are given at the end of the clip. Video: https://vimeo.com/255586740 and https://www.youtube.com/watch?v=NxCPv8Z7GU8&feature=youtu.be.

## References

[1] Cancer Research UK. Cancer incidence for all cancers combined, http://www.cancerresearchuk.org/health-professional/cancer-statistics/incidence/all-cancers-combined (accessed 1 September 2017).

[2] Loeb S Vellekoop A Ahmed HU et al Systematic review of complications of prostate biopsy. Eur Urol 2013; 64: 876–892. 23787356 10.1016/j.eururo.2013.05.049

[3] Rosario DJ Lane JA Metcalfe C et al Short term outcomes of prostate biopsy in men tested for cancer by prostate specific antigen: prospective evaluation within ProtecT study. BMJ 2012; 344: d7894. 22232535 10.1136/bmj.d7894 PMC3253765

[4] Anastasiadis E van der Meulen J Emberton M Hospital admissions after transrectal ultrasound-guided biopsy of the prostate in men diagnosed with prostate cancer: a database analysis in England. Int J Urol 2015; 22: 181–186. 25257575 10.1111/iju.12634

[5] Batura D Gopal Rao G The national burden of infections after prostate biopsy in England and Wales: a wake-up call for better prevention. J Antimicrob Chemother 2013; 68: 247–249. 23047808 10.1093/jac/dks401

[6] Mistry M Parkin DM Ahmad AS et al Cancer incidence in the United Kingdom: projections to the year 2030. Br J Cancer 2011; 105: 1795–1803. 22033277 10.1038/bjc.2011.430 PMC3242594

[7] Carignan A Roussy JF Lapointe V et al Increasing risk of infectious complications after transrectal ultrasound-guided prostate biopsies: time to reassess antimicrobial prophylaxis? Eur Urol 2012; 62: 453–459. 22575912 10.1016/j.eururo.2012.04.044

[8] Han M Chang D Kim C et al Geometric evaluation of systematic transrectal ultrasound guided prostate biopsy. J Urol 2012; 188: 2404–2409. 23088974 10.1016/j.juro.2012.07.107 PMC3876458

[9] Barringer B Carcinoma of the prostate. Surg Gynecol Obstet 1922; 34: 168–176.

[10] Grummet JP Weerakoon M Huang S et al Sepsis and ‘superbugs’: should we favour the transperineal over the transrectal approach for prostate biopsy? BJU Int 2014; 114: 384–388. 24612341 10.1111/bju.12536

[11] Galfano A Novara G Iafrate M et al Prostate Biopsy: The Transperineal Approach. European Association of Urology and European Board of Urology. EAU-EBU Update Series 2007; 5: 241–249.

[12] Bass EJ Donaldson IA Freeman A et al Magnetic resonance imaging targeted transperineal prostate biopsy: a local anaesthetic approach. Prostate Cancer Prostatic Dis 2017; 20: 311–317. 28485391 10.1038/pcan.2017.13

[13] Smith JB Popert R Nuttall MC et al Transperineal sector prostate biopsies: a local anesthetic outpatient technique. Urology 2014; 83: 1344–1349. 24726315 10.1016/j.urology.2014.02.008

[14] Novella G Ficarra V Galfano A et al Pain assessment after original transperineal prostate biopsy using a coaxial needle. Urology 2003; 62: 689–692. 14550444 10.1016/s0090-4295(03)00483-7

[15] McCulloch P Altman DG Campbell WB et al No surgical innovation without evaluation: the IDEAL recommendations. Lancet 2009; 374: 1105–1112. 19782876 10.1016/S0140-6736(09)61116-8

[16] Trotti A Bentzen SM The need for adverse effects reporting standards in oncology clinical trials. J Clin Oncol 2004; 22: 19–22. 14657230 10.1200/JCO.2004.10.911

[17] Shen PF Zhu YC Wei WR et al The results of transperineal versus transrectal prostate biopsy: a systematic review and meta-analysis. Asian J Androl 2012; 14: 310–315. 22101942 10.1038/aja.2011.130 PMC3735101

[18] Dundee PE Grummet JP Murphy DG Transperineal prostate biopsy: template-guided or freehand? BJU Int 2015; 115: 681–683. 25041087 10.1111/bju.12860

[19] Wysock JS Rosenkrantz AB Huang WC et al A prospective, blinded comparison of magnetic resonance (MR) imaging-ultrasound fusion and visual estimation in the performance of MR-targeted prostate biopsy: the PROFUS trial. Eur Urol 2014; 66: 343–351. 24262102 10.1016/j.eururo.2013.10.048

[20] Wegelin O van Melick HH Hooft L et al Comparing three different techniques for magnetic resonance imaging-targeted prostate biopsies: A systematic review of in-bore versus magnetic resonance imaging-transrectal ultrasound fusion versus cognitive registration. Is there a preferred technique? Eur Urol 2017; 71: 517–531. 27568655 10.1016/j.eururo.2016.07.041

[21] Yaxley AJ Yaxley JW Thangasamy I et al Comparison between target magnetic resonance imaging (MRI) in-gantry and cognitive target transperineal or transrectal-guided prostate biopsies for Prostate Imaging-Reporting and Data System (PIRADS) 3–5 MRI lesions. BJU Int 2017; 120: 43–50. 28749035 10.1111/bju.13971

[22] Zhang Q Wang W Yang R et al Free-hand transperineal targeted prostate biopsy with real-time fusion imaging of multiparametric magnetic resonance imaging and transrectal ultrasound: single-center experience in China. Int Urol Nephrol 2015; 47: 727–733. 25820744 10.1007/s11255-015-0957-5

[23] Lian H Zhuang J Wang W et al Assessment of free-hand transperineal targeted prostate biopsy using multiparametric magnetic resonance imaging-transrectal ultrasound fusion in Chinese men with prior negative biopsy and elevated prostate-specific antigen. BMC Urol 2017; 17: 52. 28679370 10.1186/s12894-017-0241-3 PMC5499050

[24] Ma TM Tosoian JJ Schaeffer EM et al The role of multiparametric magnetic resonance imaging/ultrasound fusion biopsy in active surveillance. Eur Urol 2017; 71: 174–180. 27236496 10.1016/j.eururo.2016.05.021

[25] Recabal P Assel M Sjoberg DD et al The efficacy of multiparametric magnetic resonance imaging and magnetic resonance imaging targeted biopsy in risk classification for patients with prostate cancer on active surveillance. J Urol 2016; 196: 374–381. 26920465 10.1016/j.juro.2016.02.084 PMC5540367

[26] DiBianco J Mullins J Allaway M Ultrasound guided, freehand transperineal prostate biopsy: an alternative to the transrectal approach. Urol Pract 2016; 3: 134–140. 10.1016/j.urpr.2015.05.007 37592459

