# Supplementary material for: Improving the safety and tolerability of local anaesthetic outpatient transperineal prostate biopsies: A pilot study of the CAMbridge PROstate Biopsy (CAMPROBE) method
Source: J Clin Urol. 2018 Mar 5;11(3):192–9. doi: 10.1177/2051415818762683 (PMC5977271; doi:10.1177/2051415818762683)
Supplement: URO762683_questionnaire_1 – Supplemental material for Improving the safety and tolerability of local anaesthetic outpatient transperineal prostate biopsies: A pilot study of the CAMbridge PROstate Biopsy (CAMPROBE) method [file URO762683_questionnaire_1.pdf]

Evaluation of effects on patients undergoing routine CAMPROBE transperineal prostate biopsies

CAMPROBE Q1

Please write your initials here……………………………………….

**Date of biopsy:**	**Study number :**
**Administer immediately after biopsy**	

This questionnaire is designed to help us understand how much discomfort the procedure you have just had caused you. **For each of the questions, please place a tick in the box ✓ that most accurately indicates your experience. Please tick only one box for each question.**

1. How much **discomfort** did the initial blood test cause?

0	1	2	3	4	5	6	7	8	9

none **a great deal**

2. How much **discomfort** did the initial prostate examination (finger in the back passage) cause you?

0	1	2	3	4	5	6	7	8	9

none **a great deal**

3. How much **discomfort** did the insertion of the scanner probe cause you?

0	1	2	3	4	5	6	7	8	9

none **a great deal**

4. How **uncomfortable** was the presence of the probe in your back passage?

0	1	2	3	4	5	6	7	8	9

not at all **a great deal**

5. How much **discomfort** did the injection of local anaesthetic cause you?

0	1	2	3	4	5	6	7	8	9

none **a great deal**

6. How much **discomfort** did the actual taking of the biopsies with the needle cause you?

0	1	2	3	4	5	6	7	8	9

none **a great deal**

Perception Questionnaire

This questionnaire asks about your perceptions of the biopsy you have just had. Please answer each question by placing a tick ☐✓ in the appropriate box. Please tick only one box for each question.

7. Overall, how **painful** did you find the whole procedure?

- not at all ☐ 0
- a little ☐ 1
- somewhat ☐ 2
- a lot ☐ 3

8. Overall, how **physically uncomfortable** did you find the whole procedure?

- not at all ☐ 0
- a little ☐ 1
- somewhat ☐ 2
- a lot ☐ 3

9. Overall, how **embarrassing** did you find the whole procedure?

- not at all ☐ 0
- a little ☐ 1
- somewhat ☐ 2
- a lot ☐ 3

10. Overall, how much **loss of dignity** did you feel?

- not at all ☐ 0
- a little ☐ 1
- somewhat ☐ 2
- a lot ☐ 3

11. Overall, how much of a problem would you find having **a similar biopsy** in the future?

- not a problem ☐ 0
- a minor problem ☐ 1
- a moderate problem ☐ 2
- a major problem ☐ 3

12. Overall, if you were discussing the procedure with a friend **who requires a biopsy** in the future, how would you describe it?

- a minor procedure ☐ 0
- a moderate procedure tolerable under local anaesthetic ☐ 1
- quite a major procedure but tolerable under local anaesthetic ☐ 2
- a major procedure that requires a general anaesthetic (being put to sleep) ☐ 3
