# Supplementary material for: Improving the safety and tolerability of local anaesthetic outpatient transperineal prostate biopsies: A pilot study of the CAMbridge PROstate Biopsy (CAMPROBE) method
Source: J Clin Urol. 2018 Mar 5;11(3):192–9. doi: 10.1177/2051415818762683 (PMC5977271; doi:10.1177/2051415818762683)
Supplement: URO762683_questionnaire_2 – Supplemental material for Improving the safety and tolerability of local anaesthetic outpatient transperineal prostate biopsies: A pilot study of the CAMbridge PROstate Biopsy (CAMPROBE) method [file URO762683_questionnaire_2.pdf]

Evaluation of effects on patients undergoing routine CAMPROBE transperineal prostate biopsies

CAMPROBE Q2

Date of biopsy: **Study number:**

Please fill in 7 days after biopsy and bring to your clinic appointment

Please complete today's date ☐☐ ☐☐ ☐☐☐☐

DAY MONTH YEAR

You have recently had a prostate biopsy. We would like to get an idea as to how this may have affected you in the week or so since your biopsy.

We would like you to answer a few questions about

(a) How you are feeling at the moment

(b) How your health has been generally since you had the biopsy

(c) Urinary, sexual and bowel symptoms that you may have and how they bother you

Please answer as many questions as you can. Full instructions are given at the beginning of each section. All the answers you give will be treated confidentially. This questionnaire should take between 15 and 30 minutes to complete.

We are very grateful for your help with this questionnaire.

Feelings

Please read each item and place a tick in the box by the reply which comes closest to how you have been feeling since you had the biopsy. Don’t take too long over your replies: your immediate reaction to each item will probably be more accurate than a long thought-out response.

Tick only one box in each section.

1. I feel tense or ‘wound up’:

		A
Most of the time		3
A lot of the time		2
Time to time/occasionally		1
Not at all		0

2. I still enjoy the things I used to enjoy:

	D	
Definitely as much	0	
Not quite so much	1	
Only a little	2	
Hardly at all	3	

3. I get a sort of frightened feeling as if something awful is about to happen:

		A
Very definitely and quite badly		3
Yes but not too badly		2
A little but it doesn’t worry me		1
Not at all		0

4. I can laugh and see the funny side of things:

	D	
As much as I always could	0	
Not quite so much now	1	
Definitely not so much now	2	
Not at all	3	

5. I feel as if I am slowed down:

	D	
Nearly all the time	3	
Very often	2	
Sometimes	1	
Not at all	0	

6. I get a sort of frightened feeling like ‘butterflies’ in the stomach:

		A
Not at all		0
Occasionally		1
Quite often		2
Very often		3

7. I have lost interest in my appearance:

	D	
Definitely	3	
I don’t take so much care as I should	2	
I may not take quite as much care	1	
I take just as much care as ever	0	

8. I feel restless as if I have to be on the move:

		A
Very much indeed		3
Quite a lot		2
Not very much		1
Not at all		0

Please continue over page…

Please continue…

9. Worrying thoughts go through my mind:

		A
A great deal of the time		3
A lot of the time		2
From time to time but not too often		1
Only occasionally		0

10. I feel cheerful:

	D	
Not at all	3	
Not often	2	
Sometimes	1	
Most of the time	0	

11. I can sit at ease and feel relaxed:

		A
Definitely		0
Usually		1
Not often		2
Not at all		3

12. I look forward with enjoyment to things:

	D	
As much as ever I did	0	
Rather less than I used to	1	
Definitely less than I used to	2	
Hardly at all	3	

13. I get sudden feelings of panic:

		A
Very often indeed		3
Quite often		2
Not very often		1
Not at all		0

14. I can enjoy a good book or radio or TV programme:

	D	
Often	0	
Sometimes	1	
Not often	2	
Very seldom	3	

Hospital Anxiety and Depression Scale, Zigmond and Snaith, Acta Pschiatrica Scandinavica, 1983

General Symptoms

This questionnaire asks about some symptoms you may have experienced since you had the biopsy.

Please answer each question by placing a tick in the appropriate box.

If you are unsure about how to answer a question, please give the best answer you can.

**1.a**	Have you felt feverish **since you had the biopsy?**	Yes	☐	0
		No	☐	1
**1.b**	How much of a problem was this for you?	Not a problem at all	☐	0
		Minor problem	☐	1
		Moderate problem	☐	2
		Major problem	☐	3

**2.a**	Have you felt nauseated (sickly) or vomited **since you had the biopsy?**	Yes	☐	0
		No	☐	1
**2.b**	**How much of a problem was this for you?**	Not a problem at all	☐	0
		Minor problem	☐	1
		Moderate problem	☐	2
		Major problem	☐	3

**3.a**	Have you had pain in the area of your biopsy **since you had the biopsy?**	Yes	☐	0
		No	☐	1
**3.b**	How much of a problem was this for you?	Not a problem at all	☐	0
		Minor problem	☐	1
		Moderate problem	☐	2
		Major problem	☐	3

**4.a**	Have you felt ‘shivery’ as if you had ‘flu **since you had the biopsy?**	Yes	☐	0
		No	☐	1
**4.b**	**How much of a problem was this for you?**	Not a problem at all	☐	0
		Minor problem	☐	1
		Moderate problem	☐	2
		Major problem	☐	3

**5.a**	Have you had to contact a doctor or nurse **since you had the biopsy?**<br>(Please include any contact with NHS direct/ walk-in centre etc.)	Yes ☐	0
		No ☐	1
5.b	Was the consultation related to problems you experienced due to the biopsy?	Yes ☐	0
		No ☐	1

**6.a**	Have you been prescribed any pain-killers **since you had the biopsy?**	Yes ☐	0
		No ☐	1
6.b	Was the pain you experienced related to your biopsy?	Yes ☐	
		No ☐	

**7.a**	Have you been prescribed any antibiotics **since you had the biopsy?**<br>(Please **exclude** any tablets that were given to you to take home after the biopsy)	Yes ☐	0
		No ☐	1
7.b	Were the antibiotics given due to an infection of your bladder/urinary tract?	Yes ☐	
		No ☐	

**8.a**	Have you felt unwell in any other way we have not asked **since you had the biopsy?**	Yes ☐	0
		No ☐	1
**8.b**	**How much of a problem was this for you?**	Not a problem at all ☐	0
		Minor problem ☐	1
		Moderate problem ☐	2
		Major problem ☐	3
8.c	Were the problems you experienced related to the biopsy?	Yes ☐	
		No ☐	

**8d)**	Have you been catheterized because you couldn't pass urine **since you had the biopsy?**	Yes ☐	0
		No ☐	1
**8e)**	**How much of a problem was this for you?**	Not a problem at all ☐	0
		Minor problem ☐	1
		Moderate problem ☐	2
		Major problem ☐	3

The presence of some symptoms can bother men to a variable degree. We are interested in how you feel about the following symptoms.

**9.a**	Have you had **blood** in your **urine since you had the biopsy?**	Yes ☐	0
		No ☐	1
**9.b**	How much of a problem was this for you?	Not a problem at all ☐	0
		Minor problem ☐	1
		Moderate problem ☐	2
		Major problem ☐	3

9.c If you have had blood in your urine, how many days did it last for?

1	2	3	4	5	6	7	> 7

**10.a**	Have you had **blood** in your **motions since you had the biopsy?**	Yes ☐	0
		No ☐	1
**10.b**	How much of a problem was this for you?	Not a problem at all ☐	0
		Minor problem ☐	1
		Moderate problem ☐	2
		Major problem ☐	3

10.c If you have had blood in your motions, how many days did it last for?

1	2	3	4	5	6	7	> 7

**11.a**	Have you had **blood in your semen since you had the biopsy?**	Yes ☐	0
		No ☐	1
		Not ejaculated at all ☐	2
**11.b**	How much of a problem was this for you?	Not a problem at all ☐	0
		Minor problem ☐	1
		Moderate problem ☐	2
		Major problem ☐	3

Perception Questionnaire

This questionnaire asks about your perceptions of the biopsy you had recently. Please answer each question by placing a tick in the appropriate box. Please tick only one box for each question.

1. Overall, how **painful** did you find the biopsy procedure?

- not at all ☐ 0
- a little ☐ 1
- somewhat ☐ 2
- a lot ☐ 3

2. Overall, how **physically uncomfortable** did you find the biopsy procedure?

- not at all ☐ 0
- a little ☐ 1
- somewhat ☐ 2
- a lot ☐ 3

3. Overall, how **embarrassing** did you find the biopsy procedure?

- not at all ☐ 0
- a little ☐ 1
- somewhat ☐ 2
- a lot ☐ 3

4. Overall, how much **loss of dignity** did you feel during your biopsy?

- not at all ☐ 0
- a little ☐ 1
- somewhat ☐ 2
- a lot ☐ 3

5. Overall, how much of a problem would you find having **another biopsy** in the future?

- not a problem ☐ 0
- a minor problem ☐ 1
- a moderate problem ☐ 2
- a major problem ☐ 3

6. Overall, if you were discussing the procedure with a friend **who requires a biopsy** in the future, how would you describe it?

- a minor procedure ☐ 0
- a moderate procedure tolerable under local anaesthetic ☐ 1
- quite a major procedure but tolerable under local anaesthetic ☐ 2
- a major procedure that requires a general anaesthetic (being put to sleep) ☐ 3

Urinary symptoms

We need to find out about any urinary symptoms that you may have had since your biopsy and also how much of a problem they are. Please answer each question if possible, thinking about the **symptoms you have experienced <u>since your biopsy.</u>**

You will see that some of the questions ask about how often you have a symptom:

Occasionally = less than one third of the time

Sometimes = between one and two thirds of the time

Most of the time = more than two thirds of the time

Please put a tick in one box for each question ☑

1. Is there a delay before you can start to urinate?

- never ☐ 0
- occasionally (less than one third of the time) ☐ 1
- sometimes (between one and two thirds of the time) ☐ 2
- most of the time (more than two thirds of the time) ☐ 3
- all of the time ☐ 4

2. Do you have to strain to <u>continue</u> urinating?

- never ☐ 0
- occasionally ☐ 1
- sometimes ☐ 2
- most of the time ☐ 3
- all of the time ☐ 4

3. Would you say that the strength of your urinary stream is...

- normal ☐ 0
- occasionally reduced ☐ 1
- sometimes reduced ☐ 2
- reduced most of the time ☐ 3
- reduced all of the time ☐ 4

4. Do you stop and start more than once while you urinate?

- never ☐ 0
- occasionally ☐ 1
- sometimes ☐ 2
- most of the time ☐ 3
- all of the time ☐ 4

5. How often do you feel that your bladder has not emptied properly after you have urinated?

- never ☐ 0
- occasionally ☐ 1
- sometimes ☐ 2
- most of the time ☐ 3
- all of the time ☐ 4

6. Do you have to rush to the toilet to urinate?

- never ☐ 0
- occasionally ☐ 1

	sometimes ☐	2
	most of the time ☐	3
	all of the time ☐	4

**7. Does urine leak before you can get to the toilet?**	never ☐	0
	occasionally ☐	1
	sometimes ☐	2
	most of the time ☐	3
	all of the time ☐	4

**8. Does urine leak when you cough or sneeze?**	never ☐	0
	occasionally ☐	1
	sometimes ☐	2
	most of the time ☐	3
	all of the time ☐	4

**9. Do you ever leak for no obvious reason and without feeling that you want to go?**	never ☐	0
	occasionally ☐	1
	sometimes ☐	2
	most of the time ☐	3
	all of the time ☐	4

**10. Do you leak urine when you are asleep?**	never ☐	0
	occasionally ☐	1
	sometimes ☐	2
	most of the time ☐	3
	all of the time ☐	4

**11. How often have you had a slight wetting of your pants a few minutes after you had finished urinating and had dressed yourself?**	never ☐	0
	occasionally ☐	1
	sometimes ☐	2
	most of the time ☐	3
	all of the time ☐	4

**12. How often do you pass urine during the day?**	hourly ☐	3
	every 2 hours ☐	2
	every 3 hours ☐	1
	every 4 hours or more ☐	0

13. During the night, how many times do you have to get up to urinate, on average?

none	☐	0
one	☐	1
two	☐	2
three	☐	3
four or more	☐	4

14. Overall, how much do your urinary symptoms interfere with your life?

not at all	☐	0
a little	☐	1
somewhat	☐	2
a lot	☐	3

15. How often do you leak urine? *(Tick one box)*

never	☐	0
About once a week or less	☐	1
Two or three times a week	☐	2
About once a day	☐	3
Several times a day	☐	4
all the time	☐	5

16. We would like to know how much <u>you think</u> leaks.
How much urine do you <u>usually</u> leak (whether you wear protection or not)? *(Tick one box)*

none	☐	0
A small amount	☐	2
A moderate amount	☐	4
A large amount	☐	6

17. Overall, how much does leaking urine interfere with your everyday life?
Please ring a number between 0 (not at all) and 10 (a great deal)

0 1 2 3 4 5 6 7 8 9 **10**

not at all **a great deal**

18. When does urine leak? *(Please tick all that apply to you)*

Never – urine does not leak	☐	a
Leaks before you can get to the toilet	☐	b
Leaks when you cough or sneeze	☐	c
Leaks when you are asleep	☐	d
Leaks when you are physically active/exercising	☐	e
Leaks when you have finished urinating and are dressed	☐	f
Leaks for no obvious reason	☐	g
Leaks all the time	☐	h

IcmaleSFquestionnaire©2000 ICS/BPGstudy group; ICIQ Neurol Urodynamics 2004: 23 322-30

Sexual matters

We need to find out about any sexual symptoms or difficulties that you have and also how much of a problem they are? Please answer each question if possible, thinking about how things have been **since your biopsy.**

Office use only

How would you rate each of the following **since you had the biopsy?**

1. Your level of sexual desire?

		Office use only
Much worse than usual	☐	*1*
Worse than usual	☐	*2*
About the same as usual	☐	*3*
Better than usual	☐	*4*
Much better than usual	☐	*5*

2. Your ability to have an erection?

		Office use only
Very poor	☐	*1*
Poor	☐	*2*
Fair	☐	*3*
Good	☐	*4*
Very good	☐	*5*

3. Your ability to reach orgasm (climax)?

		Office use only
Very poor	☐	*1*
Poor	☐	*2*
Fair	☐	*3*
Good	☐	*4*
Very good	☐	*5*

4. How would you describe the usual **quality** of your erections **since you had the biopsy?**

		Office use only
None at all	☐	*1*
Not firm enough for any sexual activity	☐	*2*
Firm enough for masturbation and foreplay only	☐	*3*
Firm enough for intercourse	☐	*4*

5. How would you describe the **frequency** of your erections?

		Office use only
I **never** had an erection when I wanted one	☐	1
I had an erection **less than half** the time I wanted one	☐	2
I had an erection **about half** the time I wanted one	☐	3
I had an erection **more than half** the time I wanted one	☐	4
I had an erection **whenever** I wanted one	☐	5

6. How often have you wakened in the morning or night with an erection **since you had the biopsy?**

Never	☐	1
Less than once a week	☐	2
About once a week	☐	3
Several times a week	☐	4
Daily	☐	5

7. How often did you have <u>any</u> sexual activity **since you had the biopsy?**

Not at all	☐	1
Less than once a week	☐	2
About once a week	☐	3
Several times a week	☐	4
Daily	☐	5

8. How often did you have sexual intercourse **since you had the biopsy?**

Not at all	☐	1
Less than once a week	☐	2
About once a week	☐	3
Several times a week	☐	4
Daily	☐	5

9. Overall, how would you rate your ability to function sexually **since you had the biopsy?**

Very poor	☐	1
Poor	☐	2
Fair	☐	3
Good	☐	4
Very good	☐	5

How big a problem <u>since you had the biopsy</u>, if any, has each of the following been for you?

Office use only

10. Your level of sexual desire?

No problem	☐	*0*
Very small problem	☐	*1*
Small problem	☐	*2*
Moderate problem	☐	*3*
Big problem	☐	*4*

11. Your ability to have an erection?

No problem	☐	*0*
Very small problem	☐	*1*
Small problem	☐	*2*
Moderate problem	☐	*3*
Big problem	☐	*4*

12. Your ability to reach orgasm (climax)?

No problem	☐	*0*
Very small problem	☐	*1*
Small problem	☐	*2*
Moderate problem	☐	*3*
Big problem	☐	*4*

13. Overall, how big a problem has your sexual function been for you **since you had the biopsy?**

No problem	☐	*0*
Very small problem	☐	*1*
Small problem	☐	*2*
Moderate problem	☐	*3*
Big problem	☐	*4*

Ejaculation

The biopsy procedure may cause you to notice some changes in ejaculation or semen. Please answer each question if possible, thinking about how things have been **<u>since you had the biopsy.</u>**

1.	Have you had any sexual activity at all (including masturbation, oral or intercourse) **since you had the biopsy?**	Yes ☐<br>No ☐

			Office use only
2.	Have you noticed any blood in your semen **since you had the biopsy?**		
	None at all	☐	*1*
	Rarely	☐	*2*
	Frequently	☐	*3*
	every time you ejaculated	☐	*4*

How would you rate each of the following **since you had the biopsy?**

3.	Your ability to ejaculate (climax)?		
	Very poor	☐	*1*
	Poor	☐	*2*
	Good	☐	*3*
	Very good	☐	*4*
4.	Your satisfaction with your climax (orgasm)?		
	Very poor	☐	*1*
	Poor	☐	*2*
	Good	☐	*3*
	Very good	☐	*4*
5.	Have you noticed any pain or discomfort when you ejaculated?		
	None at all	☐	*1*
	Mild	☐	*2*
	Moderate	☐	*3*
	Severe	☐	*4*

Bowel matters

We need to find out about any bowel symptoms or difficulties that you have and also how much of a problem they are? Please answer each question if possible.

Please put a tick in one box for each question ☑

		Office use only
1. How often have you felt like you had to pass a stool (bowel movement), but did not do so **since you had the biopsy?**		
More than once a day	☐	1
About once a day	☐	2
More than once a week	☐	3
About once a week	☐	4
Rarely or never	☐	5
2. How often have you had uncontrolled leakage of stool or faeces?		
More than once a day	☐	1
About once a day	☐	2
More than once a week	☐	3
About once a week	☐	4
Rarely or never	☐	5
3. How often have you had stools (bowel movements) that were loose or liquid (no form, watery, mushy) **since you had the biopsy?**		
Never	☐	1
Rarely	☐	2
About half the time	☐	3
Usually	☐	4
Always	☐	5
4. How often have you had **blood** in your stools **since you had the biopsy?**		
Never	☐	1
Rarely	☐	2
About half the time	☐	3
Usually	☐	4
Always	☐	5
5. How often have your bowel movements been painful **since you had the biopsy?**		
Never	☐	1
Rarely	☐	2
About half the time	☐	3
Usually	☐	4
Always	☐	5

	Office use only
6. How many bowel movements have you had on a typical day **since you had the biopsy?**	
Two or less ☐	1
Three or four ☐	2
Five or more ☐	3
7. How often have you had crampy pain in your abdomen, pelvis or rectum **since you had the biopsy?**	
More than once a day ☐	1
About once a day ☐	2
More than once a week ☐	3
About once a week ☐	4
Rarely or never ☐	5
**How big a problem, if any, has each of these been for you since you had the biopsy?**	
8. Urgency to have a bowel movement?	
No problem ☐	0
Very small problem ☐	1
Small problem ☐	2
Moderate problem ☐	3
Big problem ☐	4
9. Increased frequency of bowel movements	
No problem ☐	0
Very small problem ☐	1
Small problem ☐	2
Moderate problem ☐	3
Big problem ☐	4
10. Watery bowel movements	
No problem ☐	0
Very small problem ☐	1
Small problem ☐	2
Moderate problem ☐	3
Big problem ☐	4

		Office use only
**11.** Losing control of your stools		
No problem	☐	0
Very small problem	☐	1
Small problem	☐	2
Moderate problem	☐	3
Big problem	☐	4
**12.** **Blood** in your stools		
No problem	☐	0
Very small problem	☐	1
Small problem	☐	2
Moderate problem	☐	3
Big problem	☐	4
**13.** Abdominal/pelvic/rectal pain		
No problem	☐	0
Very small problem	☐	1
Small problem	☐	2
Moderate problem	☐	3
Big problem	☐	4
**14.** Overall, how big a problem have your bowel habits been for you **since you had the biopsy?**		
No problem	☐	0
Very small problem	☐	1
Small problem	☐	2
Moderate problem	☐	3
Big problem	☐	4

Urinary matters

The following questions ask more about urinary symptoms. Some of these are similar to questions asked previously, but they are used in questionnaires in other studies. We would be very grateful if you could answer these questions as well.

Please put a tick in one box for each question ☑

		Office use only
1.	**On average, since you had the biopsy**, how often have you urinated **blood**?	
	Never ☐	*1*
	Once or twice a week ☐	*2*
	More than twice a week ☐	*3*
	Once a day ☐	*4*
	More than once a day ☐	*5*
2.	How often have you had pain or burning with urination **since you had the biopsy?**	
	Never ☐	*1*
	Rarely ☐	*2*
	About half the time ☐	*3*
	Usually ☐	*4*
	Always ☐	*5*
3.	Which of the following best describes your urinary control **since you had the biopsy?**	
	Total control ☐	*1*
	Occasional dribbling ☐	*2*
	Frequent dribbling ☐	*3*
	No urinary control whatsoever ☐	*4*
4.	How many pads per day did you use to control leakage **since you had the biopsy?**	
	No pads ☐	*1*
	1 pad per day ☐	*2*
	2 pads per day ☐	*3*
	3 or more pads per day ☐	*4*

How big a problem, if any, has each of these been for you since you had the biopsy?

Office use only

5. Dripping or leaking urine

No problem	☐	*0*
Very small problem	☐	*1*
Small problem	☐	*2*
Moderate problem	☐	*3*
Big problem	☐	*4*

6. Pain or burning on urination

No problem	☐	*0*
Very small problem	☐	*1*
Small problem	☐	*2*
Moderate problem	☐	*3*
Big problem	☐	*4*

7. Bleeding with urination

No problem	☐	*0*
Very small problem	☐	*1*
Small problem	☐	*2*
Moderate problem	☐	*3*
Big problem	☐	*4*

8. Weak urine stream or incomplete emptying

No problem	☐	*0*
Very small problem	☐	*1*
Small problem	☐	*2*
Moderate problem	☐	*3*
Big problem	☐	*4*

9. Waking up to urinate

No problem	☐	*0*
Very small problem	☐	*1*
Small problem	☐	*2*
Moderate problem	☐	*3*
Big problem	☐	*4*

		Office use only
10. Need to urinate frequently during the day		
No problem	☐	0
Very small problem	☐	1
Small problem	☐	2
Moderate problem	☐	3
Big problem	☐	4
11. Overall, how big a problem has your urinary function been for you **since you had the biopsy?**		
No problem	☐	0
Very small problem	☐	1
Small problem	☐	2
Moderate problem	☐	3
Big problem	☐	4

The 50-Item UCLA-PCI. Wei, JT, Dunn, RL, Litwin M. S *et al.*, Urology 56: 2000, 899.

Thank you very much for all your help.

International prostate symptom score (IPSS)

Are you on any medication to improve your waterworks? Yes ☐ No ☐

If yes, state the name here: ________________________

	Not at all	Less than 1 time in 5	Less than half the time	About half the time	More than half the time	Almost always	**Your score**
**Incomplete emptying**<br>Over the past month, how often have you had a sensation of not emptying your bladder completely after you finish urinating?	0	1	2	3	4	5	
**Frequency**<br>Over the past month, how often have you had to urinate again less than two hours after you finished urinating?	0	1	2	3	4	5	
**Intermittency**<br>Over the past month, how often have you found you stopped and started again several times when you urinated?	0	1	2	3	4	5	
**Urgency**<br>Over the last month, how difficult have you found it to postpone urination?	0	1	2	3	4	5	
**Weak stream**<br>Over the past month, how often have you had a weak urinary stream?	0	1	2	3	4	5	
**Straining**<br>Over the past month, how often have you had to push or strain to begin urination?	0	1	2	3	4	5	
	None	1 time	2 times	3 times	4 times	5 times or more	**Your score**
**Nocturia**<br>Over the past month, many times did you most typically get up to urinate from the time you went to bed until the time you got up in the morning?	0	1	2	3	4	5	

**Total IPSS score**	

**Quality of life due to urinary symptoms**	Delighted	Pleased	Mostly satisfied	Mixed	Mostly dissatisfied	Unhappy	Terrible
If you were to spend the rest of your life with your urinary condition the way it is now, how would you feel about that?	0	1	2	3	4	5	6

IIEF-5
(International Index of Erectile Function)

Since you had the biopsy:

1. How did you rate your confidence that you could get & keep an erection?		Very low<br>1	Low<br>2	Moderate<br>3	High<br>4	Very High<br>5
2. When you had erections with sexual stimulation, how often were your erections hard enough for penetration?	No sexual Activity<br>0	Almost Never or never<br>1	A few Times<br>2	Sometimes<br>3	Most times<br>4	Almost always or always<br>5
3. During sexual intercourse, how often were you able to maintain your erection after you had penetrated your partner?	Did not Attempt Intercourse<br>0	Almost Never or never<br>1	A few Times<br>2	Sometimes<br>3	Most times<br>4	Almost always or Always<br>5
4. During sexual intercourse, how difficult was it to maintain your erection to completion of intercourse?	Did not Attempt Intercourse<br>0	Extremely Difficult<br>1	Very difficult<br>2	Difficult<br>3	Slightly difficult<br>4	Not difficult<br>5
5. When you attempted sexual intercourse, how often was it satisfactory to you?	Did not attempt Intercourse<br>0	Almost Never or never<br>1	A few Times<br>2	Sometimes<br>3	Most times<br>4	Almost always or Always<br>5

IIEF-5-Score: ____

Thank you very much for your help.
