# Supplementary material for: Improving the safety and tolerability of local anaesthetic outpatient transperineal prostate biopsies: A pilot study of the CAMbridge PROstate Biopsy (CAMPROBE) method
Source: J Clin Urol. 2018 Mar 5;11(3):192–9. doi: 10.1177/2051415818762683 (PMC5977271; doi:10.1177/2051415818762683)
Supplement: URO762683_questionnaire_3 – Supplemental material for Improving the safety and tolerability of local anaesthetic outpatient transperineal prostate biopsies: A pilot study of the CAMbridge PROstate Biopsy (CAMPROBE) method [file URO762683_questionnaire_3.pdf]

CAMPROBE 3 point question **Patient initials:** **Date of biopsy :** **Study number :**

To be filled in conjunction with the 7 day questionnaire

Please answer these 3 questions to help us understand your experience of the CAMPROBE procedure

1. How would you compare the CAMPROBE biopsy with your previous experience of the standard transrectal ultrasound guided prostate biopsy? (Please circle a score)

0 1 2 3 4 5 6 7 8 9 10

Worse than normal biopsy (Transrectal) **Better than normal biopsy**

2. If you had to have another prostate biopsy are you more or less likely to want to have a CAMPROBE biopsy compared to a normal transrectal biopsy? (Please circle a score)

0 1 2 3 4 5 6 7 8 9 10

Less likely to want CAMPROBE **More likely to have CAMPROBE**

3. If you had a friend or relative who was about to have a first ever prostate biopsy, which method of biopsy would you recommend (please circle one option)

1. Standard transrectal biopsy

2. CAMPROBE biopsy

3. Either

Please add any comments here :____

______________ ____

____
